# Jaw Clonus: A Rhythmic Oscillatory Movement, but Not Tremor

**DOI:** 10.5334/tohm.538

**Published:** 2020-09-29

**Authors:** Laura Williams, Marie Ryan, Ronan Kilbride, Yudy Llamas-Osorio

**Affiliations:** 1 Department of Neurology, Mater Private Hospital, IE; 2 Department of Neurology, Beaumont Hospital, Beaumont, IE

**Keywords:** Jaw clonus, clonus, Jaw tremor, amyotrophic lateral sclerosis

## Abstract

**Background:**

Jaw clonus is rhythmic, oscillatory contraction of jaw muscles induced by stretch and is caused by lesions of the descending motor neurons in the corticopontine tracts.

**Phenomenology shown:**

We illustrate jaw clonus elicited with jaw activation and upon testing of the jaw jerk in a patient with amyotrophic lateral sclerosis.

**Educational value:**

This video clearly demonstrates the uncommon sign of jaw clonus, a finding which needs to be distinguished from tremor and should direct the examiner to consider lesions of the corticopontine fibres, including amyotrophic lateral sclerosis.

Hyperkinetic movements of the face and jaw are often distressing to patients and can induce pain, deformity, and anxiety. Although a hinge joint, the temporomandibular joint (TMJ) allows for complex movements. Hyperkinetic movements of the TMJ can be challenging to diagnose at the bedside but careful observation of key features can aid localisation and steer the examiner to a specific diagnosis. Here we draw attention to a rhythmic oscillatory movement of this joint, but one that is nevertheless not canonically classified as tremor.

We describe a 70year-old man who gave a three-month history of involuntary jaw movements, which interfered with speech. Additionally, he described slurred speech, difficulty swallowing and an inability to sneeze effectively. The movements occurred several times per day, particularly when walking. Examination revealed a spastic dysarthria with nasal speech and tongue fasciculations. Prominent, sustained **jaw clonus** at a rate of approximately 9.9 Hz (from video analysis) was elicited by testing his jaw jerk (Video 1) but was also present at times with simple jaw opening. Pseudobulbar affect was apparent (Video 1). There were fasciculations throughout his upper limbs along with spasticity and mild weakness with brisk reflexes throughout. Magnetic resonance imaging of brain and spine was normal. Electromyography was supportive of our clinical diagnosis of bulbar onset amyotrophic lateral sclerosis and genetic testing revealed a pathologic expansion of the hexanucleotide repeat sequence in the C9ORF72 gene.

**Video 1:** Video demonstrating jaw clonus. Here our patient describes troublesome jaw movements. We can clearly hear a spastic dysarthria. During the video he smiles, at times inappropriately, consistent with a pseudobulbar affect. Jaw clonus at a frequency of >9 Hz is elicited by testing his jaw jerk.

Video 1 demonstrates the sign of **jaw clonus**. It is uncommon but important to recognise, as it indicates central nervous system dysfunction and its identification should steer the examiner towards assessment of bulbar function and consideration of associated disorders. It should not be confused with the jaw tremor of Parkinson’s disease, essential tremor (ET) or isolated jaw tremor. **Jaw clonus** represents rhythmic, oscillatory contraction of jaw muscles induced by stretch and caused by lesions of the descending motor neurons in the corticopontine tracts. The oscillation frequency varies throughout the literature ranging from 7.5 to 15 Hz [1,2]. Higher frequencies were hypothesized to result from a shorter reflex arc, but intramuscular neuronal features and twitch profile may be influential [1,3] and explain lower frequencies also observed.

Jaw clonus may be elicited by testing the jaw jerk or by mouth opening [1], in contrast to the resting, 3–7 Hz, jaw tremor of Parkinson’s disease (PD) which is typically seen on mouth closure. Jaw tremor of ET (4–12 Hz) is generally a postural and kinetic tremor seen with voluntary mouth opening or speaking. Associated clinical features may aid diagnosis as with the patient above. For example, patients with jaw ET are more likely to have head tremor, vocal tremor and more severe arm kinetic tremor [4]. Jaw tremor may also be seen in SCA12 (cerebellar signs, 3 Hz generalised action tremor), myorhythmia (jerky, irregular, slow, constant at rest and action), and tardive jaw tremor (prior dopamine blocking drugs, co-existent tardive syndrome movements, predominantly action tremor).

In summary, we draw attention to a rhythmic oscillatory movement of the jaw, but one that is nevertheless not canonically classified as tremor. Features that differentiate it from other forms of jaw tremor were noted.
